# Clients’ satisfaction with HIV treatment services in Bamenda, Cameroon: a cross-sectional study

**DOI:** 10.1186/s12913-016-1512-5

**Published:** 2016-07-19

**Authors:** Buh Amos Wung, Nde Fon Peter, Julius Atashili

**Affiliations:** Department of Public Health and Hygiene, Faculty of Health Sciences, University of Buea, P.O. Box 63, Buea, Cameroon; Faculty of Health Sciences, University of Buea, Buea, Cameroon

**Keywords:** HIV, Clients, HIV-Services, Patient satisfaction, Cameroon

## Abstract

**Background:**

Clients have explicit desires or requests for services when visiting hospitals; inadequate discovery of their needs may result in dissatisfaction. Patient satisfaction influences retention in HIV care, adherence to HAART and serves as determinant to HIV suppression. This study’s objectives were to quantify clients’ satisfaction with HIV services in Bamenda and determine relationship between satisfaction and clients’ socio-demographic/structural characteristics.

**Methods:**

A cross-sectional study was conducted on HIV-positive patients followed-up, on treatment and who consulted in the Bamenda Regional Hospital treatment centre between July and August 2014. Participants consent was sought and data collected on client’s level of satisfaction to staff-patient-communication, staff attitudes, privacy and confidentiality and staffing and amenities situations in the hospital. Data was collected using a structured questionnaire interviewer-administered by investigator and trained health personnel. Collected data was analyzed using Epi Info version 3.5.4 and clients’ satisfaction measured using frequencies and percentages.

**Results:**

A total of 384 participants took part in this study and their median age was 37 years (IQR: 29-46). Two hundred and seventy-four (71.4 %) participants were females. Overall satisfaction with HIV services was 91.2 % and participants reported less satisfaction with overall staffing and amenities situation of the centre (3.6 %). In the multivariate analysis, only being female, employed and perceiving high number of nurses working at the treatment centre remained significant predictors of overall satisfaction with HIV services.

**Conclusion:**

A high proportion of participants expressed satisfaction with HIV services. However, some dissatisfaction is masked in this high satisfaction level. This dissatisfaction underscores need to improve staff attitudes, staff-patient-communication, employ more staff and build better patient facilities. Future studies need to focus on assessing long-term progression of satisfaction levels with services and determinants of satisfaction involving larger samples in many treatment centres.

**Electronic supplementary material:**

The online version of this article (doi:10.1186/s12913-016-1512-5) contains supplementary material, which is available to authorized users.

## Background

The HIV/AIDS pandemic is a major public health problem with an estimated 35.0 million people living with the virus globally [1]. In 2013 alone, 2.1 million people globally became newly infected and 1.5 million people died from AIDS related causes. Despite efforts to prevent new infections and deaths, there were 1.5 million new infections in sub-Saharan Africa alone with an adult prevalence rate of 4.5 % [1]. HIV prevalence in Cameroon is 4.5 % - one of the highest for the West and Central African sub region [2]. Besides this, many HIV/AIDS carriers in Cameroon are victims of a common practice of discrimination and stigmatisation and this further hinders access to treatment [3].

Again, HIV care decentralization has been enforced as a national policy in Cameroon [4] but there has been few attempts to examine the performance of this policy and particularly its role in protecting against catastrophic health expenditures [5]. The Catholic Relief Services reports that in Cameroon, follow up of people living with HIV is provider-oriented, that there is little client education and limited client involvement in clinical monitoring [6]. Furthermore, the health care system frequently experiences low access to quality drugs, inadequate staffing, coordination and management of commodities for HIV, further compounded by the fact that only 6 % of government’s budget is allocated to health [7]. This kind of services may lead to poor patient satisfaction associated with poorer HAART treatment outcomes and lower treatment adherence [8].

Patients have explicit desires for services when in hospital and inadequate discovery of their needs may result in patient dissatisfaction [9]. It is easier to evaluate patients’ satisfaction towards services received than evaluate the quality of medical services. Moreover, patient satisfaction indicators remain stable over time while clinical indicators change with technology and the pace of medical progress [10]. Thus, to make better health policies and develop measures to increase utilization of primary health care services, patients’ views and the understanding of populations’ perceptions of quality of care are very critical and important [11, 12]. Also, since one of the pillars of improving quality of health services is measuring and addressing client satisfaction, it is recommended that clinicians should avoid sidelining patients experience as too subjective or mood-oriented, divorced from the ‘real’ clinical work of measuring safety and effectiveness [13–15].

Since evaluating patients’ satisfaction should be constant so as to reformulate the baseline and to be able to assess interventions and changes in measuring care provision [16], we assessed the percentage of persons living with HIV/AIDS (PLWHA) who expressed satisfaction with overall HIV services, clients’ satisfaction with staff patient communication, staff attitudes, privacy and confidentiality, staffing and amenities and investigated the socio-demographic/structural correlates of clients’ satisfaction with HIV services in Bamenda, Cameroon.

## Methods

### Study design and setting

A cross-sectional study was conducted on adult PLWHA who were either on treatment or followed-up in the Bamenda Regional Hospital (BRH) treatment centre.

Briefly, this study was conducted in the BRH’s AIDS Treatment Centre – a centre that receives over 4000 patients per year for routine HIV-care including follow-up by doctors and other health personnel. This centre receives patients between 8 a. m and 3 p.m. from Monday to Friday. The centre has a waiting room equipped with benches where patients first wait upon arrival at the facility, a reception room where patients are registered in order of their arrival at the centre and consultation rooms where patients are consulted and counseled.

### Study population, participants and sampling

The study population was made up of attendees of the BRH AIDS treatment centre. To be eligible for the study, a participant had to be HIV positive, aged 21 or above, registered for care or taking ART in the BRH treatment centre for at least 6 months and must have been present in the centre in the month of July 2014. Patients at an advanced stage of AIDS, who were seriously ill, were mentally or physically unable to complete the study and who were unable to give their consent and provide responses to the questions in English, were excluded from the study.

Participants were enrolled in the period between July and August 2014, by two trained data collectors (perfectly bilingual nurses who were not working at the BRH) and the investigators. Each participant was eligible only once. To get a sample representative of all attendees to the centre, a list of all patients who were present in the centre in the month of July 2014 was used and balloting done to select participants. Selected participants were approached consecutively as they registered at the beginning of their visit in the centre in the month of August 2014. Once a participant agreed to participate they were given the opportunity to undergo all study enrolment processes of consenting after which they were interviewed by the trained data collection staff in an arranged consultation room in the centre either before or after their consultation without interrupting care provision services at the centre. This process continued until the calculated sample size of 384 was reached.

The sample size was determined using a formula for estimating a single population proportion for a cross-sectional study for an infinite population [17]. Since the proportion of clients who are satisfied with HIV treatment services in Bamenda and Cameroon was not known, the following assumptions were taken: proportion of poor satisfaction of 50 % at 95 % confidence level and error margin of 5 %. This gave a sample size of 384.

### Data collection

Data was collected by trained data collectors using an English version validated structured questionnaire (Additional file 1) that was used in South Africa to evaluate patients’ satisfaction with HIV and Tuberculosis treatment [18]. The questionnaire was divided into two sections: Section A on socio-demographic characteristics (sex, age, marital status, education, and employment status); and Section B with 13 questions assessing clients’ satisfaction. The responses of these 13 questions were categorical (“very satisfied”, “satisfied”, “don’t know”, “dissatisfied”, “very dissatisfied”, or “strongly agree”, “agree”, “uncertain”, “disagree”, “strongly disagree”) or binary (“yes”, “no”) and an open ended question. Though the questionnaire had been validated and used in South Africa, a pilot study was still done in the BRH treatment centre in Bamenda to assess its validity before modifying and using it in this study.

### Data management and statistical analysis

Data from the questionnaire were entered into an Epi Info 2000 database (WHO/CDC Atlanta, USA) and analysed using Epi Info version 3.5.4. Participants’ socio-demographic/structural characteristics have been described using means, medians, standard deviations and inter-quartile ranges for continuous variables and using frequencies for categorical variables.

To determine levels of satisfaction, each item in the questionnaire was separated under sub topics (global satisfaction, staff patient communication, staff attitude, privacy and confidentiality, staffing and amenities). Categorical responses in each sub topic were coded into binary variables (“Satisfied” vs “dissatisfied” and “agree” vs “disagree”). Since the percentage of “Don’t know” and “uncertain” responses was less than 10, they were considered as missing data and analysed as such. The responses to the open ended question “How do you think the services in this centre could be improved?” were coded manually into themes and a thematic analysis was done by identifying and analyzing common ideas and patterns in the responses from the data. The frequency of each response was described and each sub topic’s frequency and percentage was computed by assigning all positive satisfaction items to one newly created variable representing each sub topic.

To assess how socio-demographic/structural characteristics are associated with overall patient satisfaction, bivariate analysis and multivariate regression analysis were done on sex, age, marital status, employment status, education level and also on distance to centre, perceived number of doctors, nurses and counselors in the centre. The bivariable analysis was done by considering overall satisfaction with services as a binary outcome variable and socio-demographic/structural characteristics as predictors, then computing the odds of being satisfied with care services between participants using unadjusted Odds ratios and 95 % CI. A *P*-value ≤ 0.25 was set as the determining point for a variable to be considered as appearing to have an association with satisfaction in the bivariate analysis and be included in the multivariate logistic model [19]. The multivariate analysis consisted computing adjusted Odds ratios using a multiple logistic regression model with overall satisfaction as the binary outcome variable and patients socio-demographic/structural characteristics as predictors. All variables that had p-values <0.05 in the multivariate logistic regression model were considered as having a statistically significant association with satisfaction.

## Results

### Demographic characteristics

The characteristics of the 384 participants included in this analysis are presented in Table 1. The median age of participants was 37 years (IQR: 29-46) and a vast majority of the participants (71.4 %) were females. More than half of the participants were married (59.4 %) and 180 (47.0 %) had attained secondary school level. The median number of years of participants since they were first diagnosed HIV positive was 4 years (IQR: 2-7). Two hundred and twenty-six participants (59.0 %) were employed or had something doing which raises them income and the median distance from their homes to the treatment centre was 5 km (IQR: 3-9). The mean perceived number of counselors working in the BRH treatment centre was 6 (SD: 1.70) while that for doctors and nurses was 1 (SD: 0.37) and 5 (SD: 0.89) respectively.

**Table 1:** Characteristics of the overall study population (N (%) or Mean (SD) or Median (IQR))

Characteristic	N or Mean or Median	% or SD or IQR
Age	37	29-46
Sex		
Male	110	28.6
Female	274	71.4
Marital status		
Single	127	33.1
Married	228	59.4
Divorced	15	3.9
Separated	10	2.6
Never married	4	1.0
Education^a^		
Primary	122	31.9
Secondary	180	47.0
High school	66	17.2
University	15	3.9
HIV years	4	2-7
Employed		
No	157	41.0
Yes	226	59.0
Distance	5	3.00-9.00
Perceived number of Counselors	6	1.70
Perceived number of Doctors	1	0.37
Perceived number of Nurses	5	0.89

^a^Primary education involves atmost seven years, secondary at most twelve, high school at most forteen and university above forteen years of education. *N* frequency, *%* frequency in percentage, *SD* standard deviation and *IQR* inter quartile range

### Levels of satisfaction with HIV treatment services

Table 2 shows the levels of satisfaction of clients with various HIV treatment services. Almost all clients were globally satisfied with the services offered to them at the treatment centre. Three hundred and ten clients (91.2 %) reported being satisfied with the services they received. However, satisfaction levels varied greatly for some specific dimensions of quality of HIV care services.

**Table 2:** Levels of satisfaction of patients with HIV services grouped under five subtopics (*N* = 384)

Indicator	Satisfied/agree/yes
	N (%)
Overall satisfaction	
Service Satisfaction	310 (91.2)
Staff-patient communication	157 (40.9)
Staff discuss treatment with patient	377 (99.0)
Patient tells when missed taking tablet	340 (95.0)
Staff language not patient’s	123 (34.5)
Staff too busy to talk to patient	168 (53.0)
Staff attitudes	193 (50.3)
Some staff don’t respect patients	182 (54.5)
Staff I see respect me	328 (93.7)
Privacy and confidentiality	326 (84.9)
Information kept confidential at centre	309 (90.9)
Patient talks to staff in private at centre	337 (90.8)
Staffing and amenities	14 (3.6)
ARV staff preferred	
To see a nurse in a nearby clinic	59 (16.4)
To travel further to see doctor	301 (83.6)
Dirty Facility	149 (55.0)
Long queues	290 (83.8)
How to improve services	
Shorter queues	178 (58.9)
More workers	272 (90.1)
Cleaner facilities	123 (41.3)
Better patient facilities	230 (75.9)

*N* frequency, *%* frequency in percentage

- Staff-patient communication

Majority of the clients, 377 (99.0 %) agreed that the health workers (doctors and nurses) fully discuss their treatment with them and 340 (95.0 %) reported that they find it easy to tell the health workers when they missed taking their tablets. Although less than half of the participants, 123 (34.5 %) agreed that it was a problem that health workers did not speak their language, over half of the participants (53.0 %) reported that the health workers were too busy to listen to their problems anyway.

b) Staff attitudes

The attitudes of staff with regards general respect of all clients and respect of individual patients was a little contradicting. More than half of the participants, 182 (54.5 %) agreed that staff do not treat patients with sufficient respect, even though, a high proportion of the participants reported that the health workers they saw or have been seeing respected them.

c) Privacy and confidentiality

Three hundred and thirty-seven participants (90.8 %) reported that at the centre, they had for some times or nearly all the time been able to talk to the doctors or nurses in private and a high proportion of the clients (90.9 %) agreed that patients information is kept confidential at the treatment centre.

d) Staffing and amenities

For their ARV treatment, most clients (83.6 %) preferred doctor-based care. One hundred and forty-nine (55.0 %) of participants agreed that the facility (including waiting area and toilets) are dirty with a high proportion of clients (83.8 %) reporting that the queues to see a doctor or nurse were too long at the centre. Data from the open ended question provided some clues to the sources of dissatisfaction (shortages of drugs at the centre, the none time consciousness of staff at work, lack of female doctors to attend to females at the centre, none distribution of cotrimoxazole tablets at the centre, lack of French speakers to give health education in French, none respect of clients by some staff especially nurses, the closure of the service centre over the weekends, lack of sufficient motivation to staff to encourage them treat patients with respect and the small nature of the waiting room). Regarding the means of improving services at the centre, one hundred and seventy-eight (58.9 %) of participants reported shorter queues; a very high proportion (90.1 %) reported having more health workers; 41.3 % having cleaner facilities and 75.9 % having better patient facilities as ways of improving the services at the treatment centre. Some of the patients highlighted that coming to work early, opening centre over the weekends, offering free CD4 tests and reduced tests prizes for required examinations, giving financial aid to assist in transportation and purchase of other drugs and employing female doctors to cater for females living with HIV were also means of improving the services offered at the centre.

### Participants’ satisfaction

**Fig. 1:**
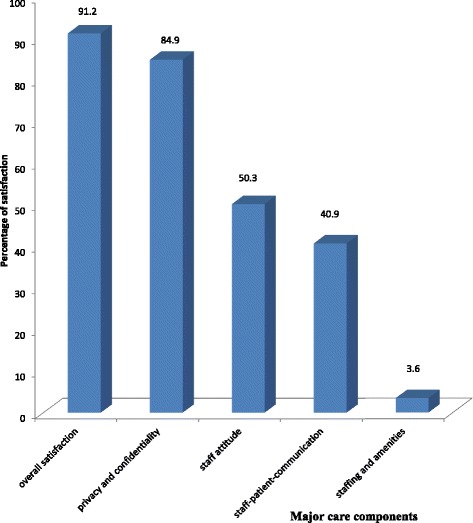
Percentage of clients’ satisfaction with major care components in Bamenda, Cameroon

Overall, after adding up positive satisfied items in each of the five subtopics of satisfaction assessments in Table 2, participants were globally satisfied (91.2 %) with the services offered at the centre. Just about half of the proportion of participants 193 (50.3 %) expressed satisfaction with staff attitudes. Three hundred and twenty six participants (84.9 %) were satisfied with the level of privacy and confidentiality at the treatment centre. However, less than half of the participants 157 (40.9 %) were satisfied with the level of staff-patient-communication and the level of satisfaction with staffing and amenities situation at the centre was relatively poor with only fourteen participants (3.6 %) being satisfied with staffing and amenities (Fig. 1).

**Fig. 2:**
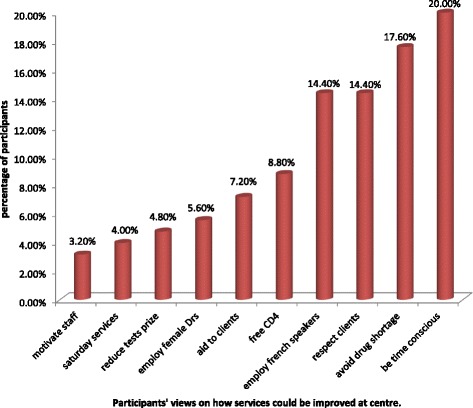
Clients’ opinions of how HIV treatment services can be improved at treatment centre in Bamenda, Cameroon

As a means to improve these services, some of the participants (20.0 %) said the staff should observe time consciousness in coming to work, 17.6 % said measures to avoid stock out or shortages of drugs at the centre should be derived and 14.4 % said respecting clients and employing French speakers to give health education in French for the benefit of francophone clients was necessary (Fig. 2).

### Correlates of clients satisfaction

The socio-demographic/structural correlates of clients’ overall satisfaction with HIV treatment services offered at this treatment centre are presented in Tables 3 and 4. In the bivariable analysis, the factors that appeared to be associated with clients’ overall satisfaction with HIV treatment services included sex, being of age 31-40 and 51 and above when compared to age 41-50, being 5-9 years since HIV positive diagnosis when compared to being less than one year since diagnosis, not being employed or not doing something that raises money compared to doing something or being employed and having increasing number of nurses at the treatment centre. In fact, the odds of being satisfied with HIV services in male participants was 0.50 times (95 % CI: 0.23, 1.09) that in female participants. The odds of being satisfied with HIV services comparing participants aged 51 and above to participants 41-50 years of age was 0.36 times (95 % CI: 0.10, 1.25). Also, the odds of being satisfied with HIV services in participants 5-9 years since HIV positive diagnosis was 0.38 times (95 % CI: 0.08, 1.80) that in participants who had not been up to a year since their HIV positive diagnosis. Again, the odds of being satisfied with HIV services in participants who said they were not employed or not doing something that raises money was 0.59 times (95 % CI: 0.28, 1.26) that in participants who said they were employed and had something doing that raises them money. Finally, the odds of being satisfied with HIV services in participants who said there are 1-4 nurses at the treatment centre was 0.50 times (95 % CI: 0.23, 1.06) that in participants who said there are 5 or more nurses at the treatment centre (Table 3).

**Table 3:** Correlates of clients’ satisfaction with overall HIV services- bivariable analysis

Characteristic	N	%	Clients satisfied with services		*P*-values
			OR^a^	95 % CI	
Age					
21-30	100	92.6	0.68	0.20-2.36	0.55
31-40	85	89.5	0.47	0.14-1.55	0.21
41-50	73	94.8	Ref	-	-
51+	52	86.7	0.36	0.10-1.25	0.11
Sex					
Male	78	86.7	0.50	0.23-1.09	0.08
Female	232	92.8	Ref	-	-
Marital status					
Single/never married	108	93.1	0.64	0.08-5.41	0.68
Married	181	89.6	0.41	0.05-3.21	0.40
Divorced/separated	21	95.5	Ref	-	-
Education^b^					
Primary	101	91.8	Ref		
Greater than primary	208	90.8	0.88	0.39-2.00	0.76
HIV years^c^					
0	42	95.5	Ref		
1-4	130	91.5	0.52	0.11-2.40	0.40
5-9	97	89.0	0.38	0.08-1.80	0.22
10+	39	90.7	0.49	0.08-2.81	0.42
Employed					
No	125	88.7	0.59	0.28-1.26	0.17
Yes	184	92.9	Ref	-	-
Distance					
1-25	282	91.6	Ref		
26-55	16	88.9	0.74	0.16-3.39	0.70
56+	12	85.7	0.55	0.12-2.61	0.45
Perceived number of counselors					
1-4	55	91.7	Ref		
5+	213	91.4	0.97	0.35-2.70	0.95
Perceived number of doctors					
1	254	90.7	0.78	0.26-2.34	0.45
> 1	50	92.6	Ref	-	-
Perceived number of nurses					
1-4	116	87.2	0.50	0.23-1.06	0.07
5+	178	93.2	Ref	-	-

^a^ *OR* unadjusted odds ratio, ^b^Primary education at most seven years and above seven years of education for greater than primary, ^c^ *HIVyrs* number of years since HIV-positive diagnosis, *OR* odds ratio, *Ref* reference variable category, *CI* confidence interval, *P*-values < 0.25 suggests possible association to satisfaction

**Table 4:** Correlates of clients satisfaction with overall HIV services- multivariable analysis

Characteristic	N	%	Clients satisfied with services		*P*-values
			aOR^a^	95 % CI	
Age					
21-30	100	92.6	0.75	0.19-2.96	0.685
31-40	85	89.5	0.44	0.13-1.53	0.198
41-50	73	94.8	Ref	-	-
51+	52	86.7	0.40	0.11-1.53	0.181
Sex					
Male	78	86.7	0.40	0.17-0.91	0.030
Female	232	92.8	Ref	-	-
HIV years^c^					
0	42	95.5	Ref		
1-4	130	91.5	0.47	0.10-2.28	0.350
5-9	97	89.0	0.32	0.06-1.67	0.176
10+	39	90.7	0.69	0.10-5.01	0.716
Employed					
No	125	88.7	0.42	0.18-0.99	0.048
Yes	184	92.9	Ref	-	-
Perceived number of nurses					
1-4	116	87.2	0.45	0.20-0.98	0.045
5+	178	93.2	Ref	-	-

^a^ *aOR* adjusted odds ratio, ^c^ *HIV years* number of years since HIV-positive diagnosis, *OR* odds ratio, *Ref* reference variable category, *CI* confidence interval, *P*-values < 0.05 are statistically significant

After adjusting for potential confounding by each of the socio-demographic/structural factors that appeared to have an association with satisfaction in the bivariate analysis, only being of female sex, being employed and perceiving increasing number of nurses in the centre remained significant predictors of satisfaction. The odds of being satisfied with HIV services in male participants was 0.40 times (95 % CI: 0.17, 0.91) that in female participants. The odds of being satisfied with HIV services comparing participants unemployed or doing nothing that raises money to those employed or doing something that raises money was 0.42 times (95 % CI: 0.18, 0.99). Finally, the odds of being satisfied with HIV services in participants who said there were 1-4 number of nurses at the centre was 0.45 times (95 % CI: 0.20, 0.98) that in participants who said there were 5 or more nurses at the treatment centre (Table 4).

## Discussion

The subjective concept of patient satisfaction is an important outcome of healthcare delivery, it can instrumentally affect health outcomes because it determines long term retention in care as well as adherence; and in scaling up HIV treatment, programme managers should not only focus on increasing number of patients on treatment to decrease HIV-related mortality but also on aspects of treatment delivery that could affect patients satisfaction [18].

In this study to appraise the level of satisfaction of clients with HIV services at the BRH HIV treatment centre, we assessed the proportion of clients who expressed satisfaction with the general services of the centre, with staff attitudes towards clients, privacy and confidentiality in handling clients and their information at the centre, staffing and amenities situation of the centre and we also assessed clients views on how services at the centre can be improved. We document that the proportion of clients satisfied with the general treatment services offered in this centre is high. Approximately, 91 % of clients are satisfied-too high a percentage per se, to raise any concerns of dissatisfaction among clients. However, dissatisfaction with particular aspects of care services such as the respect health workers show patients, waiting times, staff-patient-communication and staffing and amenities situation of the centre was also documented. In the study participants, available demographic and structural characteristics, both individually and as a group, did not accurately distinguish clients satisfied with services and those who were not. However, being of female sex, being employed and perceiving increasing number of nurses at the treatment centre had statistically significant associations with overall satisfaction.

While the overall level of satisfaction among participants appears high (91.2 %), it is within the range described among clients in similar situations and receiving similar services elsewhere. We found no studies of patients satisfaction with HIV services done in Cameroon before this study, but the levels of satisfaction with HIV services in sub-Saharan Africa has ranged from 44.3 % to 95 % and mean satisfaction ranges of 3.07 to 4.25 in Nigeria [20, 21], Ethiopia [22], Kenya [8, 23], and South Africa [18, 24]. The highest level of satisfaction (95 %) so far was detected in a study of 300 HIV-positive patients receiving treatment services in South Africa [18].

The levels of satisfaction in this study could be influenced by population characteristics or clients perceptions of adverse future variations in services if they reported being dissatisfied. Though we sampled participants in the BRH treatment centre, the sample may not have been representative of all HIV-positive patients seeking HIV services in treatment centres in Bamenda, Cameroon as the study did not include other treatment centres in the town and region at large. We do not however expect the difference in the level of satisfaction to be substantial as the BRH treatment centre offer care to a very high number of HIV-positive patients not only in Bamenda but in the whole North west region which is one of the regions with a high prevalence of HIV-positive patients in Cameroon [25]. Potential errors due to bias in data collection or poor collection of data could actually mean that our levels of satisfaction are overestimated and that our not finding an association between some socio-demographic/structural characteristics is misleading. Nevertheless, the quality of our data was assured by data collectors being trained to insure adequate data collection and the daily monitoring of the data collection process by the principal investigator.

The seemingly low levels of satisfaction observed in staff attitude towards clients (50.3 %), staff-patient-communication (40.9 %), and staffing and amenities situation of the treatment centre (3.6 %) reiterates the need for health workers to improve their attitudes towards clients, improve level of communication with clients, the need to train and employ more personnel to cater for clients and the need to build better and cleaner care facilities.

We found only one study in South Africa that assessed clients’ levels of satisfaction with HIV services and the association of clients’ satisfaction to clients’ socio-demographic/structural characteristics. Many studies in sub-Saharan Africa were conducted to establish the level of satisfaction and the association of satisfaction to socio-demographic characteristics only, without including structural characteristics of the treatment centre [8, 18, 21–24, 26]. While our sample size was adequate for estimating the levels of satisfaction with various services, only a limited number of covariates appeared to have an association with satisfaction and could be considered as potential predictors. This is similar to the study of Tran and Nguyen who found sex to have a statistical association with satisfaction with HIV services in Vietnam [27] but dissimilar with studies done in Uganda [20], Ethiopia [22] and South Africa [28, 29] which found education and socio-economic status to have an association with satisfaction. We used only overall satisfaction with HIV services as outcome variable and did not assess for the possible association of covariates to staff attitudes, privacy and confidentiality, staff-patient-communication and staffing and amenities situation of the centre. Nonetheless, we think all these are absorbed in overall satisfaction.

## Conclusion

In conclusion, the percentage of clients who expressed satisfaction with HIV treatment services at the BRH HIV treatment centre was high. The level of clients’ satisfaction with privacy and confidentiality at the treatment centre was also high. Concerning staff attitudes towards clients, staff-patient-communication and staffing and amenities situation of the treatment centre, the clients’ levels of satisfaction were considerably low. These low levels of satisfaction underscores the need for improvement of staff attitudes, staff-patient-communication, the need to train and employ more health personnel to manage HIV clients at the centre and the need to build better spacious and cleaner HIV treatment facilities. There was a relationship between client satisfaction and only three clients’ socio-demographic/structural characteristics (being female, being employed and perceiving increasing number of nurses working at the treatment centre), other factors that could possibly contribute to clients’ satisfaction needs to be explored. A prospective study of the long term progression of client satisfaction levels with HIV services right from initiation of treatment and the determinants of satisfaction, involving a sufficiently larger sample of HIV-positive patients in many different treatment centres is needed for further guidance in policy making.

## Abbreviations

AIDS, acquired immune deficiency syndrome; aOR, adjusted odds ratio; ART, antiretroviral therapy; ARV, antiretroviral drugs; BRH, Bamenda Regional Hospital; CI, confidence interval; HAART, highly active antiretroviral therapy; HIV, human immune deficiency virus; IQR, Interquartile range; IRB, Institutional Review Board; N, frequency; OR, odds ratio; PLWHA, people living with HIV/AIDS; Ref, reference variable; SD, standard deviation; WHO, World Health Organisation

## Additional file

Additional file 1: Patient satisfaction questionnaire. (DOCX 36 kb)

## References

[1] Kaiser Family Foundation. The global HIV/AIDS epidemic. Available at: http://www.kff.org/global-health-policy/fact-sheet/the-global-hivaids-epidemic/. Accessed 05 Sept 2014.

[2] Index Mundi. Cameroon HIV/AIDS-adult prevalence rate-demographics. Available at: http://www.indexmundi.com/cameroon/hiv_aids_adult_prevalence_rate.html. Accessed 05 Sept 2014.

[3] IRIN. Enduring HIV stigma in Cameroon. Available at: http://www.irinnews.org/Report/99519/Enduring-HIV-stigma-in-Cameroon. Accessed 20 Sept 2014.

[4] Roux P, Kouanfack C, Cohen J, Marcellin F, Boyer S, Delaporte E, Carrieri P, Laurent C, Spire B, Stratall ANRS 12110/ESTHER Study Group (2011). Adherence to antiretroviral treatment in HIV-positive patients in the Cameroon context: promoting the use of medication reminder methods. J Acquir Immune Defic Syndr 1999.

[5] Boyer S, Abu-Zaineh M, Blanche J, Loubiere S, Bonono R-C, Moatti J-P, Ventelou B (2011). Does hiv services decentralization protect against the risk of catastrophic health expenditures? Some lessons from Cameroon. Health Serv Res.

[6] Ngang PN, Chingang LC, Muko KN, Mokom OC (2013). From provider-oriented to client-driven care: report on the development and testing of a take-home monitoring tool for people living with HIV in Cameroon.

[7] Management Sciences for Health. Cameroon. Available at: http://www.msh.org/our-work/country/cameroon. Accessed 05 Sept 2014.

[8] Vo BN, Cohen CR, Smith RM, Bukusi EA, Onono MA, Schwartz K, Washington S, Turan JM (2012). Patient satisfaction with integrated HIV and antenatal care services in rural Kenya. AIDS Care.

[9] Tateke T, Woldie M, Ololo S (2012). Determinants of patient satisfaction with outpatient health services at public and private hospitals in Addis Ababa, Ethiopia. Afr J Prim Health Care Fam Med.

[10] Anand D, Kaushal SK, Gupta SC (2012). A study on status of client satisfaction in patients attending government health facilities in Agra District. Indian J Community Health.

[11] Peltzer K (2009). Patient experiences and health system responsiveness in South Africa. BMC Health Serv Res.

[12] Murti A, Deshpande A, Srivastava N (2013). Service quality, customer (patient) satisfaction and behavioural intention in health care services: exploring the Indian perspective. J Health Manag.

[13] Doyle C, Lennox L, Bell D (2013). A systematic review of evidence on the links between patient experience and clinical safety and effectiveness. BMJ Open.

[14] Corodeanu D-TA, Copoeru I (2013). Evaluating patient satisfaction – a matter of ethics in the context of the accreditation process of the Romanian hospitals. Procedia - Soc Behav Sci.

[15] Asefa A, Mitike G (2014). Prevention of mother-to-child transmission (PMTCT) of HIV services in Adama town, Ethiopia: clients’ satisfaction and challenges experienced by service providers. BMC Pregnancy Childbirth.

[16] Merkouris A, Athini E, Hatzimbalasi M, Rovithis M, Papastavrou E (2013). Assessment of patient satisfaction in public hospitals in Cyprus: a descriptive study. Health Sci J..

[17] Naing L, Winn T, Rusli B (2006). N. Practical issues in calculating the sample size for prevalence studies. Arch Orofac Sci.

[18] Chimbindi N, Bärnighausen T, Newell M-L (2014). Patient satisfaction with HIV and TB treatment in a public programme in rural KwaZulu-Natal: evidence from patient-exit interviews. BMC Health Serv Res.

[19] Bursac Z, Gauss CH, Williams DK, Hosmer DW (2008). Purposeful selection of variables in logistic regression. Source Code Biol Med.

[20] Nabbuye-Sekandi J, Makumbi FE, Kasangaki A, Kizza IB, Tugumisirize J, Nshimye E, Mbabali S, Peters DH. Patient satisfaction with services in outpatient clinics at Mulago hospital, Uganda. Int J Qual Health Care. 2011;5:mzr040. 10.1093/intqhc/mzr040 21775313

[21] Okoye MO, Ukwe VC, Okoye TC, Adibe MO, Ekwunife OI (2014). Satisfaction of HIV patients with pharmaceutical services in South Eastern Nigerian hospitals. Int J Clin Pharm..

[22] Belay M (2013). HIV⁄AIDS Patients’ satisfaction on ART laboratory service in selected governmental hospitals, Sidamma Zone, southern Ethiopia. Sci J Public Health.

[23] Odeny TA, Penner J, Lewis-Kulzer J, Leslie HH, Shade SB, Adero W, Kioko J, Cohen CR, Bukusi EA (2013). Integration of HIV care with primary health care services: effect on patient satisfaction and stigma in Rural Kenya. AIDS Res Treat.

[24] Magoro MT, Hoque ME, Heever HVD (2011). ART patients’ satisfaction level regarding comprehensive HIV and AIDS care management and antiretroviral treatment programme in Pretoria. South Afr J Infect Dis.

[25] Denis & Lenora Foretia Foundation. HIV/AIDS in Cameroon – An update. Available at: http://www.foretiafoundation.org/category/public-health-topics/. Accessed 25 Aug 2014.

[26] Nwabueze SA, Adogu POU, Ilika AL, Asuzu MC (2013). Comparative analysis of patient satisfaction levels in HIV/AIDS care in secondary and tertiary health care facilities in Nigeria. Afrimedic J.

[27] Tran BX, Nguyen NPT (2012). Patient satisfaction with HIV/AIDS care and treatment in the decentralization of services delivery in Vietnam. PLoS One.

[28] Jacobsen K, Hasumi T (2014). Satisfaction with healthcare services in South Africa: results of the national 2010 General Household Survey. Pan Afr Med J..

[29] Myburgh NG, Solanki GC, Smith MJ, Lalloo R (2005). Patient satisfaction with health care providers in South Africa: the influences of race and socioeconomic status. Int J Qual Health Care.

