# Supplementary material for: Clients’ satisfaction with HIV treatment services in Bamenda, Cameroon: a cross-sectional study
Source: BMC Health Serv Res. 2016 Jul 19;16:280. doi: 10.1186/s12913-016-1512-5 (PMC4950718; doi:10.1186/s12913-016-1512-5)
Supplement: Additional file 1: — Patient satisfaction questionnaire. (DOCX 36 kb) [file 12913_2016_1512_MOESM1_ESM.docx]

## Patient satisfaction questionnaire

Dear respondent, thank you for accepting to participate in this study. The following statements are some things people say about medical care. Please listen attentively; keeping in mind the care you are receiving in this centre. (We would like to know how you feel about the quality of your care here. Your answers to the questions will help us understand what works and what doesn’t, and how we can improve services rendered to you and others). We are very interested in your feelings Good or Bad, about the medical care you have received here.

Data collector number……… Participant’s code……….

1. Socio-demographic/structural characteristics
2. Your age………………………..years
3. Sex (please tick): 0 = Male..., 1 = Female………
4. Marital status (select one)

0=Single…,1=Married…, 2=Devorced…, 3=Separated…,4=Never married…

1. Level of education (select one)

0=Primary…, 1=Secondary…, 2=High school…, 3=University…

1. How long have you been followed up since your HIV diagnosis……years/……..months
2. Are you employed or doing something that gives you money? 0=No…, 1=Yes
3. What is the distance from your house to this centre……..Kilometers
4. How many counselors are in this centre…..
5. How many doctors are in this centre…….
6. How many nurses are in this centre……
7. Measures of the indicators of patient satisfaction with healthcare
8. How satisfied are you with the services in this centre? (select one)

0=Very satisfied…, 1=Satisfied…; 2=Dissatisfied…, 3=Very dissatisfied…; 4=Don’t know…

Please indicate how strongly you AGREE or DISAGREE with each of the following statements. (Tick only one, where appropriate).

|  |  | Strongly agree | Agree | Uncertain | Disagree | Strongly disagree |
| --- | --- | --- | --- | --- | --- | --- |
| 2 | The doctors and nurses (health workers) discussed the treatment fully with me |  |  |  |  |  |
| 3 | I find it easy to tell the health workers when I have missed taking my tablets |  |  |  |  |  |
| 4 | It is a problem that health workers do not speak my language |  |  |  |  |  |
| 5 | The health workers are too busy to listen to my problems |  |  |  |  |  |
| 6 | Some staff do not treat patients with sufficient respect |  |  |  |  |  |
| 7 | The health workers I see respect me |  |  |  |  |  |
| 8 | Patient information is kept confidential in this clinic |  |  |  |  |  |
| 9 | The facilities (including waiting area and toilets) are dirty |  |  |  |  |  |
| 10 | The queues to see a doctor or nurse are too long at this facility |  |  |  |  |  |
| 11 | In this clinic you able to talk to the doctors or nurses in private |  |  |  |  |  |

12) For your ARV treatment, what would you prefer (select one)

0=To see a nurse in a nearby clinic…

1=To travel further to see doctor…

2=Don’t know (indifferent)…

13. How do you think the service in this clinic could be improved?

1. Shorter queues 0=No…1=Yes…..
2. More Heath workers 0=No…1=Yes….
3. Cleaner facilities 0=No…1=Yes….
4. Better patient facilities (toilets, waiting room area etc) 0=No…1=Yes…
5. Don’t know 0=No…1=Yes….
6. Others specify………...…………………………………………….………………………

……………………………………………………………………………………………………………………………………………………………………………………………

Thank you for your time!

Source: adapted from Chimbindi Natsayi, Bärnighausen Till and Newell Marie-Louise. Patient satisfaction with HIV and TB treatment in a public programme in rural KwaZulu-Natal: evidence from patient-exit interviews. BMC Health Services Research 2014, 14:32
